# 66108 Central Cholinergic Synapse Formation in Optimized Primary Septal “Hippocampal Co” cultures

**DOI:** 10.1017/cts.2021.411

**Published:** 2021-03-30

**Authors:** Sarra Djemil, Claire R. Ressel, Amanda K. Schneeweisi, S. Abdel-Ghani, Daniel T.S. Pak

**Affiliations:** Georgetown University

## Abstract

ABSTRACT IMPACT: Optimization of primary septal-hippocampal co-cultures facilitates studying central cholinergic synapse formation and dysfunction OBJECTIVES/GOALS: Septal cholinergic innervation to the hippocampus is critical for normal learning and memory and is severely degenerated in Alzheimer’s disease. To understand the molecular events underlying this loss, we optimized a primary septal-hippocampal co-culture system that facilitates the study of central cholinergic synapses. METHODS/STUDY POPULATION: We developed an optimized in vitro septal-hippocampal co-culture system modified from previously published protocols. Briefly, hippocampal and septal tissue were harvested from embryonic day 19 (E19) Sprague-Dawley rats, digested with 0.1% trypsin, and an equal number of cells from each region plated onto coverslips coated with poly-D-lysine and laminin at a final density of 300 cells/mm2. We use immunostaining with validated primary antibodies and a fluorescent binding assay, together with confocal microscopy, to determine the structure of cholinergic synapses that are 1) native, 2) mammalian, 3) CNS derived, 4) comprised of physiological synaptic partners, and 5) developmentally mature. RESULTS/ANTICIPATED RESULTS: After DIV21, co-cultures maintained a healthy morphology. A subpopulation of neurons strongly expressed the cholinergic markers vesicular ACh transporter (vAChT), choline acetyltransferase (ChAT), and the high-affinity choline transporter (ChT1), whereas most neurons lacked vAChT expression and were presumably glutamatergic or GABAergic. The percentage of cholinergic neurons attained in the co-culture is ˜5-7%. The size of these cholinergic neurons is strikingly similar to that reported for BFCNs in the intact brain (mean 30μm, range 18-43μm). All sampled cholinergic neurons (28/28 neurons) also expressed molecular machinery necessary for GABA release. Staining for a cholinergic postsynaptic marker shows that 63% of the contacts made with are synaptic. DISCUSSION/SIGNIFICANCE OF FINDINGS: Primary septal-hippocampal co-cultured neurons have not been exploited extensively in the field, perhaps due to the difficulty in maintaining such cultures for extended periods. Here, we optimized an in vitro septal-hippocampal co-culture system, a powerful tool to comprehensively analyze central cholinergic synapse formation and dysfunction.

